# Hospital and Institutional News

**Published:** 1917-11-03

**Authors:** 


### HOSPITAL AND INSTITUTIONAL NEWS.

### THE TREATMENT OF SHELL-SHOCK.

IT is good hearing that Mr. Hodge, the vigorous Minister of Pensions, has at last taken in hand the thorny question of shell-shock patients. Competent medical authorities are of opinion that there are few men who have been in the fighting-line for eighteen months or more who do not manifest in some degree signs of the prolonged nervous strain to which they have been subjected, whilst the number of serious cases—*i.e.* those of men who are more or less completely incapacitated for civilian life, must already run into many tens of thousands. The scheme includes (1) special hospitals for the treatment of serious cases; (2) county centres for convalescence and re-education; (3) clinics for the supervision of men who have returned to civilian life. The organisation is to be under the direction of a prominent business man, and all questions of treatment left to a civilian specialist, or possibly a committee of medical men. On the physical side much has already been achieved by the staff of the R.A.M.C., but our increasing knowledge of shell-shock and its numerous sequelæ makes the establishment of a separate branch to deal with it a matter of the greatest urgency.

### THE HOME OF RECOVERY.

THEIR MAJESTIES THE KING AND QUEEN again demonstrated the keen interest they take in all matters concerning the welfare of those "broken in our wars" by their inspection on Friday of last week of a recently opened home for shell-shock and neurasthenic patients at Highfield, Golders Green. At one time a girls' school and more recently a home for Belgian refugees, it stands well back from the road surrounded by fifty acres or so of charming grounds, and a more restful retreat for those sent to recover from the stress of modern warfare could not be imagined. There is accommodation for 150 non-commissioned officers and men of both Services in the rambling old-fashioned house, and, in addition, there are large workshops where the men are taught such trades as carpentry, engineering, and basket-making under skilled instruction. In the grounds a number are being initiated into the mysteries of French intensive culture, with, let us hope, the best results as regards the nation's food supply. This institution is intended to serve as a model for similar institutions throughout the country, which, as mentioned elsewhere, are now being organised by the Ministry of Pensions. It is staffed by the medical officers of the Hospital for Nervous Diseases, Maida Vale, several of whom had the honour of being presented to His Majesty. There are now ninety-seven patients under treatment, and the medical reports show gratifying results.

### THE DEATH OF SIR PARDEY LUKIS.

ALL connected with Indian medical administration will learn with regret of the death of Sir C. Pardey Lukis, who has for nearly four decades been identified with public health work in India. Entering the Service from St. Bartholomew's Hospital in 1880 at the top of the list, he served in two Border campaigns, and, being transferred to the civil branch, he held various appointments in the United Provinces, and became Civil Surgeon of Simla in 1899, and later surgeon to the Viceroy. In 1905 he was appointed Principal and Professor of Medicine in the Calcutta Medical College, and five years later became Director-General, a position which he held continuously till his death, this in point of time constituting a record. He was an acknowledged authority on tropical medicine, and the author of several works upon that and allied subjects. Sir Pardey was in no sense implicated in the early breakdown of medical arrangements for the Mesopotamia campaign, since the executive responsibility rested with the Director of Medical Services (invariably an R.A.M.C. officer), although when the scandals became known he stepped into the breach and did what was possible to retrieve the irretrievable.

### RED CROSS AEROPLANES.

IN battle there are many cases of wounds which are likely to prove fatal unless they can receive operative treatment at once, and yet after any extensive military action the congestion of the communications will prevent the bringing of the wounded back to the hospitals as rapidly as theory requires. For cases of this nature, Dr. Chassaing, the Deputy for the Puy de Dôme, has suggested that the aeroplane offers a speedy method of avoiding the congested roads, and that it could be employed for carrying the more urgent cases back to the hospitals. It is further suggested that as a protection the aeroplanes should have the red cross painted on their wings; we are by no means sure that this marking would in any way ensure their immunity from the attacks of the enemy. There is something in the idea of this use of aeroplanes for carrying urgent cases; but in practice it must always be very restricted. Nearly three years ago something of the kind was suggested; but the aeroplane was then intended to be employed in carrying aid to the wounded, not in bringing the wounded to hospital. It was even proposed that an operating table might be carried to the field of battle, with operator, anæsthetic, dressings, and instruments. We fear that this could only be done in those cases where the field of battle remains in our hands. The idea of attempting to operate in "No Man's Land" is, we fear, beyond the possibility of realisation while our enemies are as they are.

### SPECIAL CONSTABLES' GIFT OF AMBULANCES.

WHEN the final history of the war comes to be written not least in honour will rank the Special Constabulary of London. Their duties are arduous, and to men who are for the most part well past the prime of life must be doubly so. In these days of air-raids, and the consequently more exacting duties which they impose upon members of the Force, it is all the more creditable to learn of the magnificent donation, amounting to close upon £20,000, which they have just made to the War Office for the purchase of motor ambulances for the Front. On Saturday afternoon a detachment of 4,000 of the members of the W (Brixton) Division paraded on Tooting Common to hand over the first instalment of ten fully-equipped cars. The sum of £4,421, with which they were purchased, has been entirely subscribed by this Division. When the donation is complete forty-five cars will have been presented for the use of the wounded. General Croft Atkins, who accepted the cars on behalf of the War Office, referred to the extraordinarily good feeling that existed between the Special Constabulary and our fighting men. Certainly a more generous and practical form of sympathy it would be hard to find.

### CIVILIAN DOCTORS AND THE R.A.M.C.

There has been considerable dissatisfaction in certain circles at the summary dismissal of a number of civilian doctors in military hospitals by the Medical Council at the War Office. A committee of the doctors who forwarded a protest to Lord Derby have received a reply in which the latter emphasises the point that their discharge was to be in no way regarded as a dismissal in the strict sense of the word, but rather as an attempt to supply the tremendous demand for practitioners in civilian life. The entry of the United States into the conflict has made available the services of a large number of American medical men, who will in increasing numbers be used to replace our own civilian doctors. Lord Derby expressed his regret at the short notice they had been given, which, in view of the somewhat exceptional circumstances then obtaining, seemed to him imperative. He desired to extend to all the civilian practitioners, on behalf of himself and the Army, his most sincere thanks for the services they had rendered to the nation in its hour of need.

### OZONE FOR WOUNDS.

Of the discovery of treatments there is no end, and our knowledge of the technique and possibilities of wound cleansing has advanced so much during the present war as to make us wonder how on earth a wound healed at all before August 1914! The latest therapeutic agent is ozone, and so impressed are the War Office authorities by its efficacy that they have commissioned Mr. Quain, its introducer, a well-known Ottawa electrical engineer, to equip many of the London military hospitals with his own special generating apparatus. The special advantages of this treatment are most apparent in the sterilisation of old-standing cavities of the femur and tibia. These, as everyone knows, under all other known forms of treatment were liable to run a very chronic course, and long-lasting sinuses, coupled with lameness, were only too often the result of a wound in these situations. In a recent article in the *Lancet* Major Stoker gives the results of this new treatment in seventy-nine cases, which, he says, "cannot be regarded as anything but satisfactory from every standpoint, humanitarian, scientific, or economic." The results of the ozone, which has a wonderful healing effect, are, as far as one can say at present, three : (1) It causes an increased blood-flow to the affected part; (2) it is a germicide, rapidly destroying all pathogenic organisms; (3) according to the researches of the French chemist Hennocque, it assists greatly in the formation of hæmoglobin. Mr. Quain's apparatus is, we understand, very light and portable, and can be worked either from an electric-light circuit or by accumulators. It will be interesting to see the results of the treatment, but it would be unwise to expect too much.

### AN UNDENOMINATIONAL "CHAPLAIN" SUGGESTED.

The Saffron Walden Board of Guardians will probably have decided by the time that these lines are in print whether or not to appoint another chaplain. Whatever the decision, the issue is clear. The present chaplain, the Rev. L. Hughes, Vicar of Saffron Walden, has replied to a request of the Guardians that he cannot permanently continue to act as honorary chaplain to the infirmary. He points out that though the staff of the parish church has acted for many years as chaplains without remuneration to the hospital and almshouses, its members cannot indefinitely extend the area of gratuitous service, especially since a Sunday service at the Union has involved the parish church of late in the expense of securing outside help for its own services. The chairman, the Rev. H. G. B. Smith, is in favour of the appointment of a chaplain at a salary of £40 per annum, and the board is limited to a priest of the Church of England. It has been suggested that, in place of a chaplain, "an instructor in religious knowledge" should be appointed, whose suitability should be judged by the Local Government Board. Since a chaplain is essential to a properly administered infirmary and union, and since the Guardians' choice is limited by law or precedent to a Church of England chaplain, Mr. Smith's view is the right one, nor can he be justly accused of a denominational bias in the matter. That Mr. Hughes has consented to "carry on" till Christmas, and that his arguments are reasonable, will have proved, we hope, sufficient to lead the board to attach a salary to the post of chaplain.

### INCURABLES' HOSPITAL TO CHANGE ITS NAME.

At the annual meeting of the Royal Hospital for Incurables, Putney Heath, held yesterday (after we had gone to press), the agenda contained a proposition to change the name of the hospital to "The Royal Hospital and Home for Incurables." There must always be a certain danger of confusion in respect of the names of the two great homes for incurables, and the new title, although better descriptive of the work of the charity, comes even a little nearer to that of the British Home and Hospital for Incurables. At the same meeting an election of in-patients and pensioners is included in the proceedings. For the twenty-five vacancies there are 155 applicants, and consequently 130 candidates and their friends will be disappointed. Of course, no hospital or home can relieve every person who applies, however eligible or deserving the case may be, but in the instance of these voting charities there is something suffered beyond mere disappointment; every candidate cannot hope for success unless a campaign is entered upon involving much labour and expense to himself and friends, who, in the event of non-success, generally prepare themselves for another weary struggle after a short interval. The war will alter many things, and now is the time for great changes. We believe that if the voting system were done away with not a soul would mourn its departure, and the charities affected would not in the long run be financial losers.

### ARUNDEL EMERGENCY HOSPITAL.

The Arundel and District Emergency Hospital has just entered upon a new life: the townspeople of the little Sussex borough have decided to take over the responsibility which, since the inauguration of the institution in 1906, has been mainly borne by the Duchess of Norfolk. Her Grace founded the hospital, but now, owing to the death of the Duke, and the war, she finds herself unable to promise the same financial support which has come from the Castle heretofore. The suggestion to close the institution for the duration of the war could not be thought of: such a course would have been nothing short of a catastrophe, besides being a slight upon the generosity of the Duke and Duchess of Norfolk during its eleven years' existence. The estimated expenditure is about £700 a year, and there is an old-standing deficit of £300; but with the very generous help from the ducal family still forthcoming, though not on such a large scale—the young Duke, not yet in his teens, giving £200 a year, and her Grace £20, in addition to paying the rates and taxes—the townspeople have certainly acted very wisely in the decision at which they have arrived. But their action must be supported by a substantial increase all round in the subscription list, and there is no more satisfactory way than in an increased number of subscribers. The financial burden must not be left to the few, as is so often the case with such institutions. It must be brought home to every inhabitant in the district that without their support—and support commensurate with their means—the hospital cannot be carried on. As to the medical and nursing sides of the hospital work, it has only to be noted that during the year just ended sixty-one out of sixty-six patients were cured, four were relieved, and only one died; no further words are necessary.

### DANGER IN SWIMMING BATHS.

In a recent number of The Hospital (September 22, 1917, page 505) we commented on a report of a method for purifying the water in swimming baths, whereby any change of water was claimed to be unnecessary. Somewhat akin to this subject is an occurrence which has recently taken place at Sheffield. There an inquest was held on a girl thirteen years of age, who had died from meningitis, said to have been due to middle-ear disease. After attending a swimming gala, she complained of pains in the head and vomited; she was taken to the Royal Hospital, where she died. Evidence was given by an assistant schoolmistress to the effect that the water in the bath was dirty, and that it was impossible to see the bricks on the bottom of the bath. On the other hand, the manager of the baths said that the colour of the water was due to the heavy rains which had fallen recently, and so the water was peaty. The water was changed twice a week in winter and three times a week in summer. In their verdict, the jury expressed the opinion that the disease of the middle ear was possibly the result of infection received in swimming at the baths. It is, of course, of the first importance that all the care possible should be taken to have the water in public swimming baths as free from microbic infection as possible, but we see no harm in the water being stained with peat, as so often happens when the water is collected from moors. The evidence as to the infectivity of the water was inconclusive. It is, however, certain that in many public baths sufficient care is not taken to maintain the water in a perfectly clean condition. We are expressing no opinion that the water in the Sheffield baths was unsatisfactory. With regard to the question of the origin of the meningitis from infection in the baths, it is in the highest degree unlikely that any direct infection from the water occurred. Apparently the child had disease of the middle ear, and this of itself is quite capable of causing meningitis. It is possible that in diving the increased pressure caused by the sudden submersion may have driven the septic material in the middle ear more deeply, so that the infection spread to the brain, but this might have occurred even though the water had been perfectly pure.

### KISS-IN-THE-RING AND DIPHTHERIA.

In the urban district of Paul, in the west of Cornwall, there has been, even from the beginning of the year, no small number of cases of diphtheria, one, two, or even three cases being notified every other week or so, although the total population is only a little over 6,000. This frequency of cases lasted for six or seven months, and then suddenly the number of new cases amounted to ten in a week, and this occurred after a gala festival. Dr. Russell Phillips, the medical officer of health, put forward the suggestion that this sudden increase was due to the game of Kiss-in-the-Ring having been played at the gala. The suggestion is not unreasonable, though it seems to have been received with surprise by some members of the local council, who said that the outbreak was much more likely to be due to bad water, bad milk, or bad drainage; if these defects exist, they should certainly be speedily remedied. When so many cases in all have occurred in the district during the year, it is probable that several "carriers" have existed, and the kissing may well have served to spread the disease. With regard to the existence of so much diphtheria in the district, it would surely be worth while to make a systematic examination of the throats of the children in the schools to discover the "carriers" present. If this were done, it is probable that the endemic character of the disease might cease.

### SCHOOL MEDICAL SERVICES.

EVEN during the war School Medical Services must not be neglected, and the Board of Education has expressed the hope that education authorities will use their best endeavours to secure the maintenance of the essentials of such service by obtaining the help of medical substitutes—women or older men, whole time or part time—or by other means. In this matter, the Middlesex County Council, which before the war had three assistant school medical officers, proposes to appoint three temporary medical assistants, the salary being £350 per annum each. Dr. Winifred Warner, who was an assistant school medical officer under the Middlesex County Council, has just resigned to take an appointment under the London County Council. The Middlesex Council has just appointed Miss A. Gauntlett, Mrs. E. French, and Miss J. A. Kiernan as school nurses, each at a salary of £100 a year.

### THE CHILDREN OF THE MINE-SWEEPERS.

THE Royal Naval and Marine Orphan Home at Portsmouth, which was recently visited by the Commander-in-Chief, Admiral the Hon. Sir Stanley C. Colville, who presided at the annual meeting, is caring for a larger number of children than in any previous year. The number of girls in the home is 163, and fifty boys are at Swansea on the funds of the Home. The grant from the Trafalgar Fund has amounted to the record sum of £6,238, and a splendid donation of £4,000 has been received from the War Pensions and Statutory Committee. This is part of a sum sent by the people of Canada. Such generous gifts have enabled the committee to meet the future with hope and confidence. Two new departures have been made by sending girls who show special ability to secondary schools, in order that they can be trained for suitable callings, and by maintaining boys until the age of sixteen, with the view of their being trained for sea service. What our sailors and marines have done is beyond praise, and among them we include the mine-sweepers. The dauntless gallantry and fearless deaths of our sailors and marines form a glorious record of devotion. Appreciation of such heroism can be shown in no more practical way than in providing help for their orphans.

### THE FIRST ANNUAL REPORT.

CERTAINLY one of the most tastefully printed and illustrated reports we have seen for some time is that of the Leicester West-End Association for the Entertainment of Wounded Soldiers. It is the first annual report of the Association, and includes accounts made up to September 30, 1917. At first, on regarding the amount of excellent paper used in the issue, and the number of portraits and illustrations produced by half-tone process, we were inclined to suspect a certain amount of extravagance out of keeping with war-time, but, whether the economic justification may be sound or not, the advertisers, who have taken up thirty-two pages, must have paid the better part of the printing and postage bill. The Association has supplied war hospitals in the County of Leicester with many things in the shape of libraries, magic-lanterns, pianos, billiard-tables, and comfortable furniture of all kinds, that must have made the lot of many of our wounded defenders easier and happier. A large number of teas, entertainments, matinées, and athletic sports have in addition been organised. The Association is a very live one, and the name of the honorary secretary, Mr. J. I. American, has an atmosphere of alertness which is in keeping with this attractive first annual report.

### TRAINING THE INTERNED.

A REPORT has just been made to the Committee of the Red Cross on the progress of the joint societies' scheme for the institution of training courses for interned prisoners in Switzerland. Arrangements have been made, under the direction of Major Robert Mitchell, of the Ministry of Pensions, and Lord Sandwich, for the training of men in piano-work, leather-work, carpentry, military tailoring, motor mechanism, etc. The main feature sought after has been to provide, as far as possible, training in trades where employment would be assured to men on their return to England, and such assurance has been obtained in practically all the courses organised. Major Charley, who, when himself interned in Switzerland, was largely responsible for the organisation of the work, has consented to return and superintend its progress at Vevey, Murengen, and Seeburg. We understand that Lord Sandwich will shortly proceed to Holland to make a survey of conditions there, with a view to starting a similar system of training.

### EXCESSIVE PRESCRIBING IN DERBYSHIRE.

A PERIODICAL grievance of the panel doctor has again cropped up in this county, where twenty-one medical men are to be surcharged £454 10s. for excessive prescribing in 1914. It is one of the strangest anomalies of the existing insurance system that medical men should be penalised, one might almost say in direct ratio to the efficiency of the services they render. There is at the present time much dissatisfaction amongst patients at the treatment they receive at the hands of their insurance doctor. "Panel stuff is no good," is a common complaint amongst the working classes, and it is certainly a grave matter if the doctor is limited by financial considerations to prescribing drugs which are in many cases the merest *placebos*. Amongst the doctors themselves there is a strong movement afoot to demand an increase of payment per insured person. In spite of the rise of all necessities they are receiving exactly the same as before the war, a sum which was barely sufficient in times of peace and is now totally inadequate.

In the event of the establishment of a Ministry of Health, the Insurance Commissioners will have to revise their position very carefully, if they do not wish to yield to the claims of a National Medical Service.

### HONOURS WON BY SO-CALLED CRIMINALS.

The Philanthropic Society's Farm School, Redhill, is an institution that dates from the year 1788. It is meant for the reception, education, and reformation of young boys of the criminal classes, of whom 300 are received. Most of these do well in after-life, which shows that their faults are mostly due either to bad surroundings or to the high spirits of youth. Particularly good is the record of the school in regard to National Service. According to the last report there were 761 boys serving as sailors or soldiers, of whom 181 have joined up since the war began. A complete record of what all these have done is not attainable, but news of some has come to their old school, and the following distinctions have been gained:—Six Mentions in Dispatches, seven awards of the D.C.M. (one twice), eleven Military Medals, three Russian Crosses of St. George, one Military Cross, and six commissions from the ranks, while a considerable number hold various degrees of non-commissioned rank. Not a bad record for boys who might under other circumstances have become a misery to themselves and a misfortune to their country. The Warden, in giving this report of his old boys, mentioned with satisfaction that none of them were "conscientious objectors." Evidently patriotism and self-sacrifice are among the lessons taught at the Redhill Farm School.

### WAGES IN LIEU OF HOLIDAYS.

At the Wandsworth County Court Judge Harrington had to deal with an unusual claim against the Metropolitan Asylums Board by a temporary attendant at the Tooting Bec Asylum, who claimed 37s. in lieu of holidays. He had been at the asylum from October 1914 to December 1916, when he resigned, but in October 1916 he had a week's holiday, though after his period of service he maintained he was entitled to a fortnight's holiday; but he was informed by the clerk to the Board that owing to his voluntary resignation he had forfeited his right to the money in lieu of the second week's holiday. Owing to the war a large number of officers had had no holidays, but they were paid in lieu of holidays. On behalf of the managers it was maintained that the attendant had lost his right to compensation because he left before the end of the year, though he made a request in October for the full holiday. Had he remained till February he would have been entitled to it, though probably before that he would have been asked to resign. Judge Harrington said the Board had taken care to guard themselves from undertaking any legal obligation to pay compensation. It was purely a matter for their discretion. He did not see how it was possible for him to interfere with that discretion, although he thought it had been unfairly exercised, and plaintiff ought to have been shown a little more liberality. He had a good deal of sympathy with the plaintiff, who, however, had not made out any legal right. He gave judgment against him, but made no order as to costs.

### THE SOUTH WALES NURSING ASSOCIATION.

Through the efforts of the Marchioness of Bute over £5,000 has been raised as an endowment fund for the South Wales Nursing Association. There are districts in the great coalfields where the statistics of infant mortality are, to say the least, very discouraging, and the recent visit of a Local Government Board inspector who inquired into the condition of nursing in Glamorgan served to emphasise the need for many more Nursing Associations in that country. Lady Bute's endowment fund is designed to help in meeting some of these needs. The money is invested in the War Loan, and the interest is to be used in training every year five village nurses and midwives and to supplement the administrative funds of the South Wales Nursing Association, which exists for the provision and maintenance of such nurses in sparsely-populated places. The Association is co-operating with the County Councils in its endeavour to form local Associations where such are not in existence, and supplying nurses where this can be done. At a meeting of the management committee of the Association held at Swansea last week it was reported that since the last meeting six new Associations had been provided with nurses, while several other Associations are in process of formation. Lady Bute was congratulated and thanked for placing the Association in possession of the £5,000 endowment fund. Baroness de Rutzen having become joint honorary secretary of the Association, Mrs. Mount, daughter of Sir J. D. Llewelyn, was elected to succeed her as honorary treasurer. The Association is experiencing some difficulty in obtaining a sufficient number of young women for free training as village nurses.

### A GAZETTELESS HOSPITAL.

There are still a few war hospitals, even in London, which do not possess a gazette. One, for instance, is the 1st London General Hospital, Camberwell. The reason for this journalistic inertia is the familiar one of want of time for the indulgence of such trifles. Set beside this the following letter, which we have received from the matron of one of the big war hospitals which is the happy possessor of a capital gazette, of which she herself is the editor. In inviting our criticism, the matron is good enough to say, "Your expert opinion now and again is most valuable to an amateur. I know I shall never make a journalist. I am only keeping the *Gazette* alive because the staff wishes it to be kept going and cannot find anyone else to tackle it." This gazette is certainly kept going, and is it really true that the staff of the 1st London General Hospital, Camberwell, is so indifferent to amusement? We venture to assert that there is no lack of talent, and that the staff includes a painter and a sculptor. It only remains to find an editor. Existing gazettes show that these are made, not born, and that matrons as a rule can edit excellently.

### THE BIRMINGHAM DRAUGHTSMEN.

There is a spontaneity in the drawings and verses of the October number of the *Southern Cross* which gives it a high place among the gazettes of the month, and indeed among its own series. The drawing of "W. L. S.," entitled "A Medical Board," is delightfully suggestive; it gives the spirit of the occasion, because, with admirable fancy, it has thrown realism to the winds. Since, in this world, truth is told in parables while falsehood is supported by facts, the demon in the presidential chair, the waiting group of nude figures (a caricature suggestive of Blake), and the tailed Sileni with their long prongs stretched over the head of the next examinee, are admirable in spirit. It is really better than "W. L. S.'s" more serious, and less original, drawing of the 1st Southern General Hospital, Edgbaston, which forms the frontispiece. "F. M.'s" "A U.S.A. Proposition," depicting a thin figure in a kind of walking flight, also deserves mention; while the joke accompanying D. Binn's drawing is worth notice if only for the current adjective "umpteen" which is applied therein to the "inquisitive visitor's" banal question. There is spirited work, too, in Sydney R. Flower's "A Patient's Dream," one of the best drawings in this number. The joke about the man who mistook the steam-pressure indicator for a clock is not bad. We mention it to show that all the jokes and drawings have a hospital flavour. The illustrators, who are the heroes of this number, have, as is their due on this occasion, crowded out all notice of the other contents. A paragraph can give the cream, or the vinegar, of criticism only.

### THE FOURTH SOUTHERN "GAZETTE."

The monthly gazette of the 4th Southern General Hospital, Plymouth, now in the middle of its second volume, has developed in journalistic wisdom since we first had the pleasure of seeing it. Headlines, illustrations, sketches, and verse are all vigorous, but the best things have to be searched for, since the editor's modesty is such that he does not like to indulge too freely in cross-headings and other arresting devices. For instance, in the article on the late Earl of Mount Edgcumbe, whose work for this and other hospitals in war and peace was one of active interest, we find towards the end a charming little account of his lordship as a carpenter. This, by the way, can be commended to the notice of Viscount Harberton, whose amusing book, "How to Lengthen our Ears," is an attack on book-education and a defence of the fingers as the best means of improving mental growth. Lord Mount Edgcumbe, we read, "was a skilful worker, especially in carpentry. He made with his own hands many puzzles and games in woodwork for the pleasure of the wounded soldiers and sailors." Shortly before his death "he was completing a beautiful building made out of blocks of wood, and perfect in every part, as a pattern which some day might be a profit to men incapacitated through wounds from more strenuous work." How a personal touch like this gives life to that deadest of all things, the ordinary obituary notice! There is an amusing article on the fly in the trenches, and an illustration of a fly-trap, which reminds us in these cold days that the fly, if not the summer, was once among us. "Where's your blue band?" asks the gate orderly in one illustration. "Had it for breakfast," replies Tommy. The joke is new to us, at all events. It has the virtue of a hospital flavour without which any article or caricature in these gazettes is something of a false pretence. We hear that these gazettes are hard to maintain, in spite of their popularity. That they impose an immense amount of work on the editor we know. Often the busiest person in a hospital, the matron, is the editor. All honour to her. In conclusion we know how hard it is to reconcile the manager's business schemes with editorial ideas of decorum. We therefore ask if the manager *must* have so many "facing-matter" advertisements as we see in this number? The articles are interleaved with them. Good business is not always good form.

### THIS WEEK'S DRUG MARKET.

While the upward tendency of prices is, on the whole, well maintained, there are few actual changes in value of any importance; in fact quotations have reached such an extraordinarily high level that we may perhaps expect to see a slowing down in the rate of price advances. Although the limit of price beyond which consumers will refuse to do business has not yet been reached, there is little doubt that the limit has been reached, in the case of many drugs, at which business is discouraged. The Russian syndicate which controls the output of santonin has instructed its agent in London to advance the quotation for this drug. The upward tendency in the price of quinine continues, and very probably prices will be still higher. Clove oil is again dearer. Business has been done in ipecacuanha at higher rates. Salicylic acid and sodium salicylate are being produced in increasing quantities by English makers, and prices have a slightly lower tendency. Acetanilide is again dearer. The value of sandal-wood oil has an upward tendency. Supplies of chloral hydrate are far from plentiful, and there is no relief of the scarcity of cocaine.

### TO OUR READERS.

Contributions are specially invited from any of the readers of The Hospital. They should deal with topical subjects and news or incidents illustrating any department of hospital work, its progress or difficulties. They must be authenticated for the information of the Editor only. The minimum payment if published is 5s. There is no hard-and-fast rule as to space, but paragraphs of about twenty lines in length are preferred.

